# Naringin and Naringenin: Their Mechanisms of Action and the Potential Anticancer Activities

**DOI:** 10.3390/biomedicines10071686

**Published:** 2022-07-13

**Authors:** Jolita Stabrauskiene, Dalia M. Kopustinskiene, Robertas Lazauskas, Jurga Bernatoniene

**Affiliations:** 1 Department of Drug Technology and Social Pharmacy, Faculty of Pharmacy, Medical Academy, Lithuanian University of Health Sciences, Sukileliu pr. 13, LT-50161 Kaunas, Lithuania; jolita.stabrauskiene@lsmuni.lt; 2 Institute of Pharmaceutical Technologies, Faculty of Pharmacy, Medical Academy, Lithuanian University of Health Sciences, Sukileliu pr. 13, LT-50161 Kaunas, Lithuania; daliamarija.kopustinskiene@lsmuni.lt; 3 Institute of Physiology and Pharmacology, Medical Academy, Lithuanian University of Health Sciences, LT-50161 Kaunas, Lithuania; robertas.lazauskas@lsmuni.lt

**Keywords:** naringin, naringenin, flavonoids, cancer, cellular signaling pathways

## Abstract

Naringin and naringenin are the main bioactive polyphenols in citrus fruits, the consumption of which is beneficial for human health and has been practiced since ancient times. Numerous studies have reported these substances’ antioxidant and antiandrogenic properties, as well as their ability to protect from inflammation and cancer, in various in vitro and in vivo experimental models in animals and humans. Naringin and naringenin can suppress cancer development in various body parts, alleviating the conditions of cancer patients by acting as effective alternative supplementary remedies. Their anticancer activities are pleiotropic, and they can modulate different cellular signaling pathways, suppress cytokine and growth factor production and arrest the cell cycle. In this narrative review, we discuss the effects of naringin and naringenin on inflammation, apoptosis, proliferation, angiogenesis, metastasis and invasion processes and their potential to become innovative and safe anticancer drugs.

## 1. Introduction

Cancer is a complex pathology in which abnormal cells grow due to the disruption of normal proliferation and cell cycle processes, forming tumors that spread and invade other body parts [1,2]. Environmental factors, such as ultraviolet rays, pollution, radiation, smoking and stress, lead to internal changes, such as oxidative stress, impaired apoptosis and increased rates of genetic mutations [3]. Anticancer therapy is often complicated due to the ability of cancer cells to resist drugs and the therapy’s severe side effects [4].

Inclusion of greater amounts of fruits and berries, especially citrus fruits, in the human diet is suggested to help in cancer prevention and to suppress cancer growth [5,6,7]. The main active compounds of fruits are the polyphenolic compound flavonoids. In various studies, flavonoids were found to be capable of demonstrating strong anticancer effects by acting as antioxidants; modulating ROS-scavenging enzyme activity; upregulating apoptosis, autophagy and cell cycle arrest; and downregulating inflammation, proliferation processes and metastasis formation [8,9,10,11,12,13]. Naringin and naringenin, which belong to a subclass of flavonoids known as flavanones, are the main bioactive compounds in citrus fruits, and they are known for their beneficial effects on human health, which have been summarized in several recent reviews [4,14,15,16,17,18]. However, the mechanisms of the anticancer effects of naringin and naringenin have not yet been fully clarified, and they are under extensive investigation. Therefore, in this work, we provide an overview of the effects of naringin and naringenin on inflammation and cancer signaling pathways and their possible targets in tumor cells.

## 2. Chemical Properties, Pharmacokinetics and Bioavailability of Naringin and Naringenin

**Figure 1:**
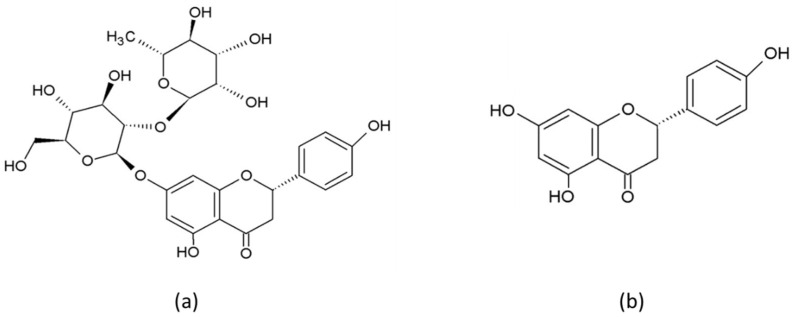
Chemical structure of naringin (**a**) and naringenin (**b**).

Naringin (4′,5,7- trihydroxyflavanone-7-rhamnoglucoside) and its aglycone form naringenin (Figure 1) belong to the flavonoid class known as flavanones and are found mainly in citrus fruits, including lemon, orange, mandarin and grapefruit [19].

The structure of flavanones differs from that of other flavonoids due to there being a chiral carbon at the C2 position and no substitution at the C3 position [17,20]. Naringin has a traditional flavonoid structure: three rings (two of them benzene rings) connected by a three-carbon chain, and two rhamnose units attached at the C7 [14,17,20]. Flavanones demonstrate potent antioxidant activity [21], which depends on the number and configuration of functional hydroxyl groups, responsible for free radical scavenging and metal ion chelating activities [22]. The 7-OH, 4′-OH and 5-OH groups, the 4(=O) carbonyl group on the C ring and the 5-OH group on the A ring are responsible for the antioxidant activity of naringenin [23], which is not as strong as that of other flavonoids due to the absence of the C2=C3 double bond [24].

The pharmacokinetic properties and bioavailability of naringin and naringenin have recently been thoroughly reviewed in [14,17]. To summarize briefly, in the human body, naringin is poorly absorbed through the gastrointestinal tract and epithelial cells of the oral cavity and of the small intestine, and intestinal microorganisms generally convert it to its aglycone form, naringenin [25]. The oral bioavailability of naringin is about 5–9% [26] at a dose of 50 mg equivalent to aglycone, whereas the C_max_ value is about 5.5 h [27]. The oral bioavailability of naringenin is around 15%, and only low amounts are absorbed in the human gastrointestinal tract due to its low solubility [28]. Naringin and naringenin are distributed in the lungs, trachea, gastrointestinal tract, liver and kidneys [29]. In the intestinal and liver cells, naringin and naringenin undergo phase I (oxidation or demethylation by cytochrome P450 monooxygenases) and phase II (glucuronidation, sulfation or methylation) metabolism [14]. Excretion is mainly through urine, and some metabolites are found also in feces. Milk proteins and bulky dietary fibers might reduce the bioavailability of naringin and naringenin [30].

Naringin is a relatively safe and nontoxic bioactive compound [31]. In commercial citrus juices, the concentration of naringin is between 50 and 1200 mg/L [32]. The oral no observed adverse effect level (NOAEL) of naringin is approximately 200 mg/kg in humans [33]. The therapeutic concentration of naringenin has been shown to be ~300 mg, taken twice daily, resulting in 8 µM of naringin in the blood, which demonstrates beneficial effects for human health [34].

## 3. Anticancer Activities of Naringin and Naringenin

At least 20% of cancers cases are associated with long-term inflammation. Chronic unregulated inflammation causes constant production of harmful ROS, which can cause DNA damage and genome changes, leading to the onset of tumor growth. On the other hand, inflammatory mediators, such as IFN-γ, TNF, IL-1α/β and IL-6, or transforming growth factors, such as cytokines and vascular endothelial growth factor (VEGF), stimulate a process improving tumor growth blood supply [35,36]. In addition, the primary inflammatory pathway, NF-κB, plays an essential role in the survival of cancer cells by allowing these cells to avoid apoptosis. Naringin and naringenin have been shown to use various mechanisms to interfere with cancer development, promotion and progression, modulating several unregulated signaling pathways associated with inflammation, proliferation, apoptosis, autophagy, angiogenesis, invasion and metastasis [37] (Table 1).

Most chemotherapeutic drugs work against cancer because they help with cell apoptosis. However, the devastating effects of chemotherapy also affect healthy cells—i.e., the gastric mucosa, hair follicles and bone marrow cells—which limits their normal functioning [36]. Therefore, toxicity is the most limiting symptom associated with chemotherapeutic agents of synthetic origin. Some therapeutically active components originating from plants may be toxic [50], while others with lower toxicity might help to increase the efficacy of typical chemotherapy treatments [51] or, due to their antioxidant and anti-inflammatory effects, might decrease or prevent tumor growth [52].

**Figure 2:**
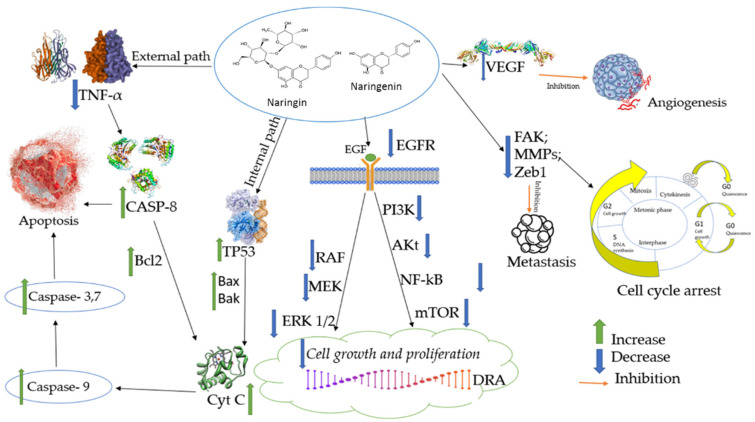
Anticancer effects of naringin and naringenin. VEGF—vascular endothelial growth factor; FAK—focal adhesion kinase, also known as PTK2 protein tyrosine kinase 2; MMPs—matrix metalloproteinases; Zxb1—gene encoding a zinc finger transcription factor that regulates the transcriptional repression of interleukin 2 [53]; EGFR—epidermal growth factor receptor; PI3K—phosphoinositide 3-kinase; NF-κB or NF-kappaB—a complex of proteins that control DNA transcription, cytokine production and cell survival; mTOR—the mammalian target of rapamycin; RAF or c-RAF—proto-oncogene serine/threonine-protein kinase, an enzyme encoded by the RAF1 gene in humans; MEK—mitogen-activated protein kinase; RAF—extracellular signal-regulated kinase; ERK1/2—extracellular signal-regulated kinase 1/2; TP53—tumor protein P53, also known as p53 and cellular tumor antigen p53; Bax and Bak—members of the Bcl-2 family and core regulators of the intrinsic pathway of apoptosis; Cyc c—the cytochrome complex, a small hemoprotein that is freely bound to the inner mitochondrial membrane, belongs to the cytochrome c protein family and plays an essential role in cell apoptosis; BID—BH3 interacting-domain death agonist, a gene and a pro-apoptotic member of the Bcl-2 protein family; Caspase-8—a caspase protein encoded by the CASP8 gene; TNF-α—tumor necrosis factor-alpha.

The anticancer potential of the flavanones, such as naringin and naringenin, has been widely discussed worldwide. Several cellular signaling pathways mediate the anti-carcinogenic activity flavanones. Recently, combination therapy integrating naringin and naringenin with current anticancer drugs has become more commonly used and demonstrated more synergistic effects than monotherapy. According to Fayung Zhang et al., naringin and naringenin can inhibit the resistance of cancer to many drugs, which is one of the most significant barriers to clinical treatment [17,21]. Inhibition of signal transduction pathways, such as vascular endothelial growth factor (VEGF) (which is capable of reducing cancer cell blood supply), FAK (PTK2); MMPS and Zxb1, reduces the formation of metastases. Overexpression of epidermal growth factor receptor (EGFR) is related to the development of a wide variety of tumors. Interruption of EGFR signaling can prevent the growth of EGFR-expressing tumors and improve the health of patients. For example, Zhao et al. reported that naringenin suppressed the migration of breast cancer cells by suspending the cell cycle at the G0/G1 phases [30]. Alternatively, the activation process—for example, that of the tumor protein P53—has been described as a “genome guardian”, and it is essential to maintain its stability by preventing mutations in the genome. In addition, sequential activation of caspases plays a vital role in cell apoptosis processes (Figure 2).

Therefore, these flavanones might have potential as bioactive compounds for use in alternative therapies to treat and prevent different types of cancers.

### 3.1. Effects of Naringin and Naringenin on Inflammation

Inflammation has an important role as the main adaptive defense mechanism against infection or injury [54]. During inflammation, macrophages produce cytokines, such as interleukin-IL, tumor necrosis factor (TNF)-α and interferon (IFN)-γ, and other inflammatory mediators, such as nitric oxide (NO) and prostaglandins (PG) [55]. Excessive production of these cytokines and anti-inflammatory mediators contributes to various inflammatory diseases, such as atherosclerosis, rheumatoid arthritis, asthma, pulmonary fibrosis and septic shock [56]. Pathogens and host-derived molecules, such as lipopolysaccharides and interferon (IFN-β), stimulate macrophages to release inflammatory mediators, such as NO, prostaglandin E2 (PGE2) and reactive oxygen species (ROS), as well as inflammatory mediators, such as inducible nitric oxide synthase (iNOS) and cyclooxygenase-2 (COX-2). Naringin and naringenin were capable of modulating the activity of human macrophages, thus reducing inflammation [57,58]. Inhibition of these inflammatory mediators is an important target when treating a disease with anti-inflammatory components [59]. Moreover, chronic diseases, such as cancer, diabetes, cardiovascular disorders, autoimmune diseases and neurodegenerative disorders, result from tissue damage and genome changes caused by persistent low-grade inflammation in and around the affected tissue or organ. Existing treatments for many chronic diseases sometimes have a more substantial effect than the disease itself; therefore, patients need safer, less toxic and more cost-effective treatment alternatives. Flavonoids and their preparations have been used for centuries to treat various human diseases, and their use has persisted to this day [35].

**Figure 3:**
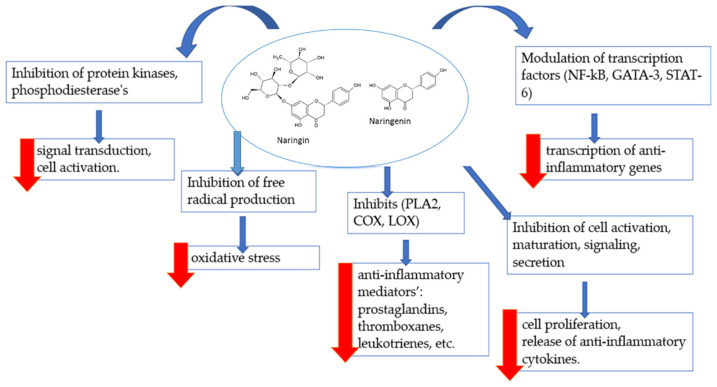
Anti-inflammatory mechanisms of flavonoids. COX—cyclooxygenase; DCs—dendritic cells; LOX—lipoxygenase; PLA2—phospholipase A2; STAT-6—signal transducer and activator of transcription-6.

Increasing scientific evidence suggests that polyphenolic compounds, such as flavonoids in fruits, vegetables, legumes or cocoa, may have anti-inflammatory properties [60]. The flavanones naringin and naringenin have various anti-inflammatory properties and act via the inhibition of regulatory enzymes [58,61,62], changes in arachidonic acid metabolism [61,62,63,64], modulation of gene expression [65], and effects on transcription factors that play essential roles in controlling mediators involved in inflammation [66]. Naringin and naringenin are also powerful antioxidants that can destroy free radicals and attenuate their formation [67]. They also significantly affect the immune cells and immune mechanisms that are important in inflammatory processes (Figure 3).

Experiments in vitro have demonstrated that naringenin significantly eliminated colitis in an induced murine colitis model. The effects of naringenin treatment could be linked, at least in part, to the inhibition of TLR4 protein and NF-kB activity, the downregulation of the expression of inflammatory mediators (iNOS, ICAM-1, MCP-1 Cox2, TNF-a, IL-6) and the inhibition of the production of inflammatory cytokines (TNF-a and IL-6) [46]. A recent study has shown the anti-inflammatory effects of hesperidin and naringin in diabetic rats. Both flavonoid compounds decreased the levels of circulating proinflammatory cytokines and downregulated the expression of IL-6 in adipose tissue [68]. The anti-inflammatory effects of flavonoids may also be attributed to their ability to bind cyclooxygenases (Coxs). Coxs catalyze the transition of arachidonic acid into prostaglandins and thromboxanes. For example, COX-2 produces prostaglandins for the induction of inflammation and pain. Studies have demonstrated the ability of flavanones to bind COX-2, which can help develop potent inhibitors for the treatment of inflammation [69].

In in vivo studies, naringin decreased airway inflammation and activated pulmonary endothelial hyperpermeability via modulation of aquaporin1 in lipopolysaccharide/cigarette smoke-induced mice [70]. In mouse models of arachidonic acid (AA)- and tetradecanoylphorbol-13-acetate (TPA)-induced ear edema, naringin and naringenin exerted topical anti-inflammatory and anti-allergic activities [64]. Furthermore, in a rat model of cyclophosphamide-mediated hepatotoxicity, naringin decreased oxidative stress, fibrosis and inflammation [71]. In a model of 1,2-dimethyhydrazine (DMH)-induced precancerous lesions in the colons of Wistar rats, naringenin decreased lipid peroxidation, ROS generation, lesion formation and levels of TNF-α [72]. Thus, flavanones could act as anti-inflammatory agents in various in vitro and in vivo models of inflammation.

### 3.2. Effects of Naringin and Naringenin on Autophagy

Macroautophagy, or simply autophagy (ATG), is an essential “self-eating” process that cells perform to allow degradation of intracellular components, including soluble proteins, aggregated proteins, organelles, macromolecular complexes and foreign bodies [73]. Autophagy is primarily a cytoprotective mechanism [74]. However, excessive self-degradation can be deleterious. Meanwhile, autophagy dysfunction is associated with various human pathologies, including cancer, aging and metabolic diseases, such as diabetes and lung, liver and heart diseases [75].

**Figure 4:**
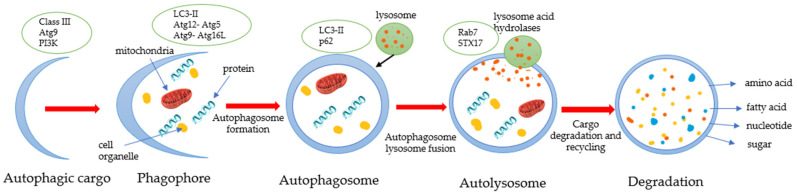
Process of autophagy. Class III PI3K mediates the production of PI3P, a key lipid-signaling molecule required for autophagosome formation.

External stimuli, such as nutrient deficiency, hypoxia, cytokines, hormones, DNA damage, and mTOR inhibition, lead to inducement of autophagy (Figure 4).

Autophagy characterizes anticancerogenic effects in normal cells and inhibits malignant cell transformation. However, degeneration of autophagy is associated with gene disorders, cellular metabolism, tumor-immune care, invasion and metastasis and tumor drug resistance [76]. Therefore, drugs that target autophagy can act as antitumor drugs. The mechanisms of flavonoids’ regulation of autophagy vary across different tumor cells. Multiple flavanones regulate the autophagy of tumor cells by targeting the mTOR signal pathway or stimulate autophagy by targeting apoptosis-related proteins or HMGB1(HMGB1 is secreted by immune cells, such macrophages, monocytes and dendritic cells) to regulate the interaction between Bcl-2 and Beclin-1 [77]. Blc-2 family proteins control the release of cytochrome c during mitochondrial dysfunction. Beclin-1 can be upregulated to activate the autophagy pathway using autophagy-related genes (ATG) and protein products. The essential step in autophagosome formation is the cleavage of LC3. Naringenin increases the level of protein LC3 and the expression of ATG5, Beclin-1 and p62 and has shown significant results in osteosarcoma treatment [76].

Previous studies have confirmed that autophagy is an essential signal downstream of the PI3K/AKT/mTOR pathway that is involved in drug-induced cancer cell apoptosis [78]. It activates autophagy by inhibiting the PI3K/AKT signal, thereby inhibiting the growth of gastric cancer cells. The protective effects of naringin and naringenin result from the activation of the PI3K-Akt-mTOR pathway and the inhibition of autophagy [78]. Furthermore, studies have demonstrated that naringin has an impact on autophagosome formation. Previous research showed that naringin induced autophagy by increasing Beclin-1 protein, including converting cytosolic LC3-I protein to autophagic isoform LC3-II [45].

Therefore, flavanones cause cancer cell death by inhibiting autophagy through signaling pathways, which significantly impacts the further treatment of tumors, especially in combination with other chemical preparations.

### 3.3. Effects of Naringin and Naringenin on Apoptosis

Apoptosis, also known as “self-killing” is a form of type I programmed cell death. The intracellular death program is activated when the cells are no longer needed. Apoptosis sustains cell populations and is associated with tissue growth, development and aging [79]. Apoptosis is also appears a defense mechanism under pathological conditions. For example, when cells are too damaged to recover, they experience apoptosis through caspase-dependent and -independent mechanisms.

**Figure 5:**
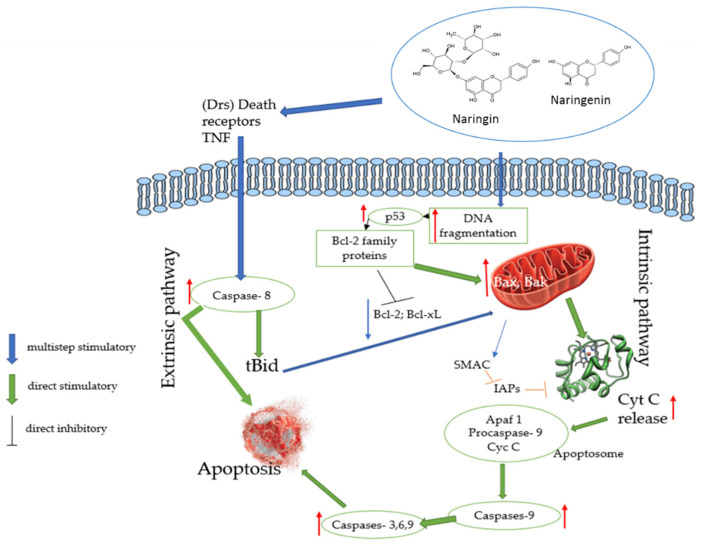
Naringin and naringenin targets in extrinsic and intrinsic apoptosis pathways. Red arrows show the effects of flavonoid activation and blue the effects of flavonoid suppression. The intrinsic or mitochondrial apoptosis pathway is initiated by releasing cytochrome c (Cyt c) to the mitochondria, which binds to apoptotic protease activating factor 1 (Apaf-1), and procaspase-9 to form the apoptosome. Then, caspase-9 is cleaved, and effector caspases are activated. The extrinsic or cytoplasmatic apoptotic pathway is activated at the cell surface by binding a specific ligand to its corresponding cell surface death receptor. Death receptors promote the recruitment of adapter proteins, which can interact with caspase-8 to generate its active form. Caspase-8 can also interact with the intrinsic apoptotic pathway by splitting pro-apoptotic proteins (tBid), which results in cytochrome c release. Anti-apoptotic members of the Bcl-2 family (B-cell lymphoma protein 2 (Bcl-2) and Bcl-2 homolog splice variants (Bcl-xL)) can block apoptosis, but its pro-apoptotic members (Bax and tBid) can also regulate programmed cell death. Other mitochondrial proteins can inhibit both initiator and effector caspases [81].

Apoptosis mainly consists of two main pathways—extrinsic and intrinsic. Extrinsic pathways are triggered by external stimuli or ligand molecules and involve death receptors (DRs). The intrinsic pathway is mediated by Bax/Bak insertion into the mitochondrial membrane. Subsequently, Cyc c is released, which combines with Apaf-1 and procaspase-9 to produce apoptosome, and this is followed by the activation of caspase-3,6,9 cascades of apoptosis. Epidemiological studies have clarified the beneficial effects of dietary polyphenols (flavonoids) in reducing the risk of chronic diseases, including cancer [9,80]. Cancer cells are resistant to apoptosis, which is a form of programmed cell death commonly caused by signal transduction pathways, pro-apoptotic proteins, caspases and Bcl-2 family proteins. During the last few years, it has been shown that flavonoids can cause apoptosis by modulating several essential elements in cellular signal transduction pathways linked to apoptosis (caspases and Bcl-2 genes). In addition, flavanones such as naringin and naringenin have shown great potential as cytotoxic anticancer agents, promoting apoptosis in cancer cells [9] (Figure 5).

In the results from across different studies, flavanone naringenin could induce apoptosis through increased p53 expression, induced Bax and caspase-3 cleavage, downregulated Bcl-2 and survived in the SGC-7901 cell line. In addition, the naringenin-induced extrinsic apoptotic pathway was related to the over-expression of TNF- family proteins [82]. Furthermore, it has been reported that naringenin inhibited the migration of breast cancer MDA-MR-231 cell lines via modulation of inflammatory and apoptotic signaling pathways [43]. Finally, naringenin also inhibited the migration and invasion of glioblastoma cells due to inhibition of ERK and p38 activities [83]. In conclusion, both naringin and naringenin have potent effects on apoptotic actions.

### 3.4. Effects of Naringin and Naringenin on Proliferation

**Figure 6:**
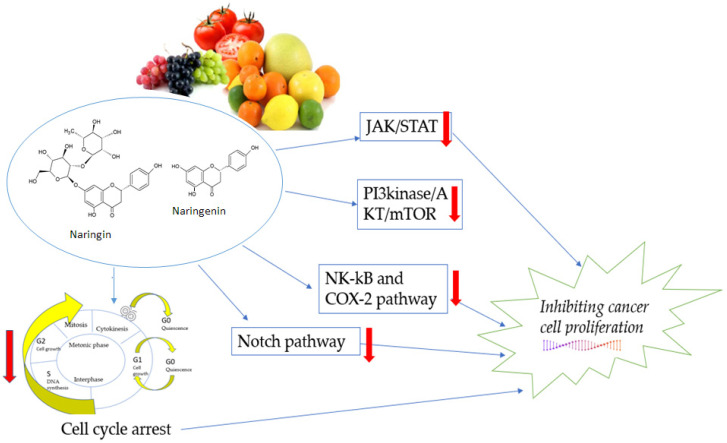
The primary mechanisms of flavanones in the proliferation pathway. JAK—Janus kinase, a family of intracellular, non-receptor tyrosine kinases that transduce cytokine-mediated signals via the JAK-STAT pathway; STAT—transcription factors; PI3K—phosphatidylinositide 3-kinase; AKT—protein kinase B, a significant mediator of cell survival; mTOR—the mammalian target of rapamycin; NF-κB—nuclear factor-kappa-light-chain-enhancer of activated B cells; COX-2—cyclooxygenase 2; Notch—the Notch signaling pathway, a highly conserved cell signaling system present in most animals.

Cell proliferation is a crucial process in homeostasis, and cell development is tightly regulated to ensure specific genome duplication. Loss of cell cycle control leads to the proliferation of cancer cells [84]. Targets such as the JAK/STAT, PI3K/Akt and mTOR, Notch, NK -kB and COX-2 signaling pathways are essential for the regulation of various cytokines and growth factors that affect many essential cellular functions and promote cell proliferation, growth and differentiation, as well as migration, inflammation, immune response and apoptosis (Figure 6). Inappropriate signaling in the JAK/STAT pathway is associated with cancer progression and metastasis. JAK/STAT signaling is activated by interleukin-6 (IL-6). In addition, the STAT protein STAT3 can promote the proliferation of cancer cells. Inhibition of JAK1 and JAK2 kinases may reduce STAT3 activity and block its dimerization and nuclear transfer [17,85].

PI3K/Akt and mTOR pathways are important for cell proliferation, metabolism and survival under physiological and pathological conditions. PI3K enzymes are vital in activating the PI3K/Akt/mTOR pathway because they catalyze the generation of PIP3 from PIP2. Akt has many substrates that mediate cellular functions such as angiogenesis, metabolism, growth, proliferation, survival, protein synthesis, transcription and apoptosis. mTOR consists of two distinct functional complexes, mTORC1 and mTORC2, which are involved in the metastasis cascades of cell growth, proliferation, motility, survival, invasion and migration. Akt activates mTOR through at least two mechanisms, namely direct activation or indirect activation [44,47]. 

The Notch signaling cascade is essential for cell proliferation, differentiation, development and homeostasis, and abnormal Notch signaling is associated with various cancers, such as prostate, breast, colon, and lung cancers, and T-cell leukemia central nervous system malignancies [86].

NF-κB proteins are a group of rapidly acting primary transcription factors that control a wide range of cellular processes, such as inflammatory and immune responses, developmental processes and cell growth, proliferation, survival and apoptosis. These transcription factors are activated by various stimuli, including cytokines, free radicals, bacterial and viral infections, UV radiation and carcinogens. In addition, NF-κB is a significant regulator of COX-2 expression, acting as a transactivator of the COX-2 promoter, and is involved in the activation of COX-2 in cancer cells [36,55].

Cancer is characterized by uncontrolled proliferation and an impaired cell cycle, leading to abnormal invasion and metastasis [2,9]. Cancer cells are characterized by various mutations that ignore antiproliferative signals and, thus, contribute to proliferative growth. Meanwhile, flavonoids have a wide variety of anticancer effects: they modulate ROS-scavenging enzyme activities, contribute to arresting the cell cycle, induce apoptosis autophagy and suppress cancer cell proliferation and invasiveness. Furthermore, flavonoids act as pro-oxidants and may suppress proliferation of cancer cells through the inhibition of epidermal growth factor receptor and mitogen-activated protein kinase (EGFR/MAPK), as well as phosphatidylinositide 3-kinase (PI3K), protein kinase B (AKT) and nuclear factor-kappa-light-chain-enhancer of activated B cells (NF-κB). For example, in various tumor cell types, flavanone naringenin has a strong inhibitory effect on the PI3k/AKT/mTOR signaling pathway [47]. The results of previous studies have demonstrated that naringin inhibited the proliferation of CRC cells in a dose-dependent manner. In addition, naringin promoted the apoptosis of CRC cells and inhibited the activation of the PI3K/AKT/mTOR signaling pathway in a dose-dependent manner [87]. 

Based on the evidence obtained in one study, it was found that one of the flavanones, naringenin, could inhibit the proliferation of an HT-29 colon cancer cell line at concentrations of 0.71–2.85 mM [85]. Furthermore, Kawaii et al. found significant antiproliferative activity in naringin and naringenin at concentrations >0.04 mM for all four cancer cell lines studied. In addition, naringin was a weaker cell proliferation inhibitor than its aglycone form naringenin [2].

A growing number of studies provide evidence that naringin and naringenin inhibit cell proliferation, migration and invasion and increase apoptosis of cancer cells in in vitro and in vivo models of cancer, therefore demonstrating substantial anticancer effects on several types of human cancer, such as bladder, hepatocellular, breast, colorectal and gastric cancers [66].

### 3.5. Effects of Naringin and Naringenin on Angiogenesis

Neoangiogenesis is required for tumor development and progression. Vascular proliferation occurs in many solid tumors due to the production of angiogenic factors, especially vascular endothelial growth factor [88]. Mice with subcutaneous gliomas treated with naringin (120 mg/kg/day) were assayed using the endothelial HUVEC cell line for tube formation and migration and demonstrated suppressed tube formation and reduced cell invasion [89]. Furthermore, in an in vitro model, naringin at 0.1 µmol/L inhibited vascular endothelial growth factor release from MDA human breast cancer cells and from U-343 and U-118 glioma cells [88]. Malignant melanoma is one of the most deadly skin cancers due to its aggressive proliferation and metastasis [90]. In vitro and ex vivo angiogenesis assays demonstrated that naringenin treatment potently suppressed endothelial cell migration, tube formation and sprouting of microvessels in a dose-dependent manner in B16F10 and SK-MEL-28 cells [90]. In a human endothelial cell model, naringenin treatment suppressed angiogenesis in vitro, as evaluated by proliferation, apoptosis, migration and tube-formation assays [91]. The chick chorioallantoic membrane (CAM) assay showed that naringenin also inhibits physiological angiogenesis in vivo, reducing CAM neovascularization [91].

### 3.6. Effects of Naringin and Naringenin on Metastasis and Invasion

Cancer cells are able to invade local tissues via an invasion process and migrate from their original sites to distant ones, where they establish new tumors via the metastasis process [92]. Once a cancer spreads, it is harder to eliminate it [93]. 

Naringin (10 or 20 μM) suppressed proliferation and invasion in human osteosarcoma MG63 cells by inhibiting zinc finger E-box binding homeobox 1, a transcriptional repressor of epithelial differentiation involved in tumor metastasis, resulting in downregulation of cyclin D1 and matrix metalloproteinase 2 (MMP-2) [94]. Naringin (5–20 μM) suppressed invasion and adhesion of human glioblastoma U87 cells, as assessed by the Matrigel transwell, cell adhesion and wound-healing assays [52]. A gelatin zymography assay and Western blot analyses demonstrated that its mechanism of action was related to the decreased enzymatic activities and protein levels of MMP-2 and MMP-9, and it also reduced the protein phosphorylation of extracellular signal-regulated kinase (ERK), p38 mitogen-activated protein kinase and c-Jun N-terminal kinase [52]. At noncytotoxic concentrations (3–30 μM), naringin downregulated vascular cell adhesion molecule-1 (VCAM-1) by increasing miR-126, thus suppressing the migration and invasion of the cells in chondrosarcoma, a primary malignant bone cancer that is highly invasive and tends to form distant metastases, especially in the lungs [95].

Naringenin at 500 μM inhibited human two-pore channel 2, thus inhibiting the progression and reducing the metastatic potential of melanoma [96]. Naringenin (20–160 μM) suppressed the cell migration and cell invasion tendencies of MDA-MB-231 breast cancer cells, as assessed by a transwell assay [97]. Transforming growth factor β (TGF-β) has been shown to promote tumor invasion and metastasis by activating the MMPs, although the signaling mechanisms controlling this process have not yet been fully clarified [92]. Combined therapy with a Smad7 agonist—asiatic acid (10 mg/kg/day intraperitoneally (i.p.) for 4 weeks)—and a Smad3 inhibitor—naringenin (50 mg/kg/day i.p. for 4 weeks)—restored the balance between Smad3 and Smad7 signaling in the TGF-β-rich tumor microenvironment and significantly suppressed tumor invasion and metastasis in mouse models of melanoma and lung carcinoma [92]. Naringenin (100 and 200 µM, applied for 48 h) reduced the expression of MMP-2 and MMP-9, thus decreasing human lung cancer proliferation, migration and metastasis in vitro [98]. In a Boyden chamber analysis, 100, 200 and 300 μM naringenin reduced migration and invasion of cells in glioblastoma, a brain cancer characterized by high invasion and drug resistance [93]. In another study, 100, 200 and 300 μM naringenin suppressed the activities of MMP-2 and MMP-9, as well as the ERK and p38 signaling pathway, in glioblastoma cells, thus preventing metastasis formation [93]. Naringenin (20, 40 and 80 μM) downregulated MMP-2 and MMP-9 and subsequently inhibited migration in gastric cancer SGC-7901 cells [43]. Naringenin (50 µM and 100 µM) blocked TGF-1/SMAD3 downstream signals, reduced the expression of mesenchymal markers and attenuated MMP-2 and MMP-9 activities, consequently suppressing migration and invasion in pancreatic cancer panc-1 and aspc-1 cells [99]. Naringenin (300 µM over a period of 24 h) decreased AKT and MMP-2 activities and inhibited migration of TSGH-8301 bladder cancer cells [100] and proliferation of A549 lung cancer cells [40]. High concentrations of naringenin (75 μM) inhibited cell proliferation, whereas low concentrations (5 and 10 μM) decreased the motility of MAT-LyLu prostate cancer cells, which overexpress voltage-gated sodium channels that modulate their metastatic activity [101]. Naringenin and naringin at 25, 50 and 100 μM suppressed the invasiveness of the human hepatoma cell lines HepG2, Mahlavu and HA22T in a concentration-dependent manner, as assessed by transwell and wound-healing assays [102]. Thus, both naringin and naringenin can suppress metastasis and invasion of tumor cells in various cancer models in vitro and in vivo.

## 4. Conclusions and Future Perspectives

The flavanones naringin and naringenin, the main bioactive flavonoids in citrus fruits, protect against cancer and suppress proliferation processes; they thus exhibit interesting therapeutic potential for use as effective alternative remedies for oncological patients. Both compounds could be used as adjuvant therapies due to their abilities to overcome resistance to conventional chemotherapy and increase the efficacy of chemotherapeutic agents [103].

However, most investigations with pure naringin and naringenin are performed on animals or in vitro. Therefore, well-controlled trials are needed to elucidate the potential of these flavanones in clinical practice. Furthermore, there are important technological issues that must be solved to create novel formulations to improve the bioavailability of naringin and naringenin. Nevertheless, the abilities of these flavanones to decrease inflammation, promote apoptosis and inhibit proliferation, angiogenesis, metastasis and invasion processes demonstrate that they have great potential to become innovative and safe anticancer drugs.

## Figures and Tables

**Table 1:** The main anticancer effects of naringin and naringenin.

Flavanones	Pathway	Main Effect	References
***Breast Cancer***			
**Naringenin**	ERK	Inhibition of tumor growth	[38]
**Naringin**	ERK	Inhibition of cell proliferation and promotion of cell apoptosis	[39]
**Naringenin**	ROS	Inducement of apoptosis	[30]
***Lung Cancer***			
**Naringenin**	AKT/MMP	Inhibition of tumor growth and metastasis	[40]
**Naringin**	ROS	Inducement of apoptosis	[17,41]
***Gastric Cancer***			
**Naringenin**	MMP	Inhibition of chemical-induced cell invasion and metastasis	[42,43]
	ROS	Inhibition of all proliferation and inducement of apoptosis	[42]
**Naringin**	PI3K/AKT	Blocking of the PI3K/AKT pathway and activation of pro-death autophagy	[44,45]
***Colorectal Cancer***			
**Naringenin**	NF-kB/p65	Inducement of apoptosis and cell cycle arrest	[46]
**Naringin**	PI3K/AKT/mTOR	Inhibition of cell proliferation and promotion of cell apoptosis	[47]
***Prostate Cancer***			
**Naringenin**	ERK	Invasion and migration	[48]
**Naringin**	(PI3K)/AKT	Inducement of apoptosis and cell cycle arrest in G1 phase	[49]

## Data Availability

No new data were created or analyzed in this study. Data sharing is not applicable to this article.
